# Development of the Repeat Clinical Hospitalization Risk Assessment Scale in Chronic Psychiatric Diseases

**DOI:** 10.31083/AP39445

**Published:** 2025-04-01

**Authors:** Necla Şahin, Birgül Özkan

**Affiliations:** ^1^Department of Nursing, Bilkent City Hospital, 06800 Çankaya/Ankara, Turkey; ^2^Department of Psychiatric Nursing, Ankara Yildirim Beyazit University, Faculty of Health Sciences Nursing Department, 06760 Çubuk/Ankara, Turkey

**Keywords:** psychiatric nursing, repeat hospitalization, risk assessment, scale

## Abstract

**Objective:**

This study was conducted to provide a comprehensive scale that evaluates the risk of repeated inpatient hospitalizations in chronic psychiatric diseases in order to predict and prevent repeated hospitalizations.

**Methods:**

The study population consisted of individuals with chronic psychiatric diseases (n = 390) receiving inpatient treatment at the adult psychiatry inpatient services of Turkey Ankara Bilkent City Hospital. The sample number calculation was made based on 10 times the number of scale items. For the pilot component of the research, data was collected between February, 2023 and January, 2024. An ‘Informed Voluntary Consent Form’, a ‘Sociodemographic Data Collection Form’, the ‘Discharge Readiness Scale’, and the ‘Repeated Clinical Hospitalization Risk Assessment Draft Scale for Chronic Psychiatric Diseases’ were used as data collection tools. During the development stages, the validity and reliability of the scale were analyzed.

**Results:**

The Content Validity Index (CVI) value of the scale items was calculated as 0.98. Cronbach’s alpha of the scale was found to be 0.833.

**Conclusions:**

The ‘Repeated Clinical Hospitalization Risk Assessment Scale in Chronic Psychiatric Diseases’ is a valid and reliable scale for the Turkish population in terms of measuring risk level.

## Main Points

1. The CVI value of the scale items was calculated as 0.98.

2. As a result of factor analysis, the Kaiser Meyer Olkin (KMO) coefficient was found to be 0.833.

3. The final version of the scale includes 27 items and seven sub-dimensions. The sub-dimensions are named as follows: Factor 1, ‘Social Support Resources’; Factor 2, ‘Psychosocial Functioning’; Factor 3, ‘Discharge Readiness’; Factor 4, ‘Treatment Compliance’; Factor 5, ‘Suicide Risk’; Factor 6, ‘Psychotic Feature’; and Factor 7, ‘Insight’.

4. Goodness of fit values: Root Mean Square Error (RMSEA) = 0.052, Normed Fit Index (NFI) = 0.88, Comparative Fit Index (CFI) = 0.93, Goodness of Fit Index (GFI) = 0.90, Adjusted Goodness of Fit Index (AGFI) = 0.87, and χ^2^/sd = 2.042. Cronbach’s alpha of the scale was found to be 0.833.

5. The ‘Repeated Clinical Hospitalization Risk Assessment Scale in Chronic Psychiatric Diseases’ (RCHRASCPD) is a valid and reliable scale for the Turkish society in terms of measuring risk level.

## 1. Introduction

One in every eight people in the world experiences a mental disorder [1]. Mental disorders are classified according to the International Classification of Diseases (ICD-10) and Diagnostic and Statistical Manualof Mental Disorders, Fifth Edition (DSM-5) published by the American Psychiatric Association (APA) [2]. Chronic psychiatric diseases are mental disorders that last for a long time and often significantly affect a person’s daily life and functionality [3]. Individuals diagnosed with psychiatric diseases can be treated on an outpatient basis or can be followed up through inpatient hospitalization [4]. Individuals receive treatment in psychiatric inpatient services shortly after discharge; it is known that recurrent inpatient hospitalizations occur due to reasons such as familial and individual factors, service provided, and community structure [5, 6, 7]. The return to inpatient treatment of a diagnosed individual within a certain period of time after a previous hospitalization is termed recurrent inpatient services stay [8] and is also called the ‘Revolving Door Phenomenon’ [9].

Although the rate of readmission to psychiatric inpatient services after discharge is unknown, it decreased by 10% in 2022 and 2023, according to the National Mental Health Action Plan of the Ministry of Health of the Republic of Turkey [10].

Many studies both in Turkey and worldwide have investigated risk factors for repeat hospitalization [11]. These risk factors include insufficient social support resources [12, 13, 14], suicide attempt and risk [14, 15], being male [11, 16], early discharge [13], having psychotic symptoms [11], and treatment noncompliance [17].

As psychiatric nurses are important members of the team, they have important duties and responsibilities regarding the identification of risk groups for readmission, the risk factors associated with inpatient readmission, and the care, education, and consultancy specific to each individual in this regard [18]. Mental health nursing is a multidisciplinary field that plays a critical role in the process of protecting, developing, and improving the mental health of individuals. Nurses provide both basic and advanced interventions to individuals experiencing mental health problems [19]. Individuals who are readmitted to psychiatric inpatient services are monitored with the support of nurses from admission to discharge and follow-up in community mental health centers. In this process, nurses play a role in many areas such as improving family and social relationships [20]. Nurses play an important role in preventing repeat hospitalizations by constantly interacting with patients. It is their responsibility to meet the needs of patients and improve their quality of life by creating individualized care plans. They also strengthen patients’ social support networks and help them take their place in society by effectively using community resources [21]. As a result, nurses undertake therapeutic, educational, coordinating, and supportive roles in preventing repeat hospitalizations. In this way, patients’ quality of life increases and repeat hospitalizations, which are a burden on the health system, are reduced.

As seen in the literature, clinical data collection forms created by the study owners are used as data collection tools in studies examining the risk factors affecting recurrent clinical hospitalizations in psychiatric diseases. In order to evaluate the risk of repeated hospitalization of individuals diagnosed with chronic psychiatric diseases, we aimed to provide a comprehensive scale specific to individuals diagnosed with chronic psychiatric diseases. It is expected that the risk of repeated hospitalization of individuals diagnosed with chronic diseases who are admitted to a psychiatric inpatient service will be evaluated using this developed scale and measures will be taken to prevent readmission, thus reducing costs. We intend for the scale to be used as a data collection tool in determining the risk of repeated hospitalization during the process that begins with admission to psychiatric inpatient services. We believe that the developed scale can be applied to predict the risk of repeat hospitalization of individuals followed in community mental health centers.

## 2. Material and Methods

### 2.1 Study Design 

**Study type**: The research study type was relationship seeking.

**Place and characteristics**: The study population consisted of individuals diagnosed with a chronic psychiatric disease who received inpatient treatment at the adult psychiatry inpatient services of Ankara City Hospital in Turkey between February, 2023 and January, 2024.

**Research samples**: The sample size was determined in accordance with the criterion of using the number of samples that is 10 times larger than the number of items to be included in the planned scale. The sample of the research consisted of 390 people. The sample size was calculated before starting the study. Support was received from a statistician for this calculation. A power analysis was performed according to the sample calculation with a known universe. In addition, scale development studies in the current literature were examined [22, 23, 24, 25]. According to the common decision taken with these studies and the opinion of a statistician, a sample calculation was made at a rate of 10 times the number of items in the first version of the developed scale. Accordingly, the sample number determined according to both the expert opinion and the literature on this subject was determined as 390 people.

### 2.2 Characteristics of the Sample

#### 2.2.1 Inclusion Criteria for the Study

All individuals who were receiving inpatient treatment at the Ankara City Hospital adult psychiatry inpatient services on the specified dates, who had a diagnosis of chronic psychiatric disease, who were literate and had no obstacles to answering the questions, and who agreed to participate in the study were included in the study.

#### 2.2.2 Exclusion Criteria from the Study

Individuals who did not have a chronic psychiatric disease, individuals who did not agree to participate in the study, individuals who were illiterate, individuals with organic mental disorders, and individuals who did not have the cognitive functionality to answer questions were not included in the study.

#### 2.2.3 Withdrawal Criteria

Withdrawal criteria included incomplete research data collection forms, disruptions in the implementation process, extension of the research period due to health problems, and voluntary renunciation of participation in the research.

### 2.3 Data Collection Tools

Research data was collected using the ‘Recurrent Clinical Hospitalization Risk Assessment Scale in Chronic Psychiatric Diseases’, ‘Sociodemographic Data Form’, and ‘Readiness for Discharge Scale’.

#### 2.3.1 Draft Scale for Repeated Clinical Hospitalization Risk Assessment in Chronic Psychiatric Diseases Revised Form after Expert Opinions (Version 1)

A draft ‘Repeated Clinical Hospitalization Risk Assessment Scale in Chronic Psychiatric Diseases’ (RCHRASCPD), along with expert opinions and suggestions taken from the Expert Opinions Evaluation Form, was created with 52 items including ‘totally disagree’, ‘disagree’, ‘neither agree nor disagree’, ‘agree’, and ‘completely agree’. A 5-point Likert scale was created and used as a data collection tool during the pilot application. At this stage, the factor loadings of the items were calculated. The items with factor loadings below 0.25 were removed, but the item order was not changed.

#### 2.3.2 Draft Repeat Clinical Hospitalization Risk Assessment Scale in Chronic Psychiatric Diseases Revised Form after Pilot Implementation (Version 2)

The Repeat Clinical Hospitalization Risk Assessment Scale in Chronic Psychiatric Diseases (Version 2) was created after the pilot application. Following the analysis, the scale took its final form as 39 items and was applied to 390 individuals. The form used a 5-point Likert scale: ‘strongly disagree’, ‘disagree’, ‘neither agree nor disagree’, ‘agree’, and ‘completely agree’. Items numbered 33, 32, 34, 37, 26, 27, 25, 28, 22, 17, 20, 18, 24, and 38 were reverse scored in the form.

#### 2.3.3 Repeat Clinical Hospitalization Risk Assessment Scale in Chronic Psychiatric Diseases (Version 3)

The Repeat Clinical Hospitalization Risk Assessment Scale in Chronic Psychiatric Diseases (Version 3) is the scale created as a result of the construct validity analysis applied after data collection. It has 27 items scored on a 5-point Likert scale: ‘strongly disagree’, ‘disagree’, ‘neither agree nor disagree’, ‘agree’, and ‘completely agree’. Items 1, 2, 3, 4, 5, 6, 7, 8, 9, 10, 11, 12, 13, 25, and 26 were reverse scored. There are seven sub-dimensions in the scale. The ‘social support resources’ sub-dimension includes items 1, 2, 3, 4, and 5. The ‘psychosocial functioning’ subscale includes items 6, 7, 8, and 9. The ‘ready for discharge’ sub-dimension includes items 10, 11, 12, and 13. The ‘suicide risk’ subscale includes items 14, 15, 16, and 17. The ‘treatment compliance’ subscale includes items 18, 19, 20, and 21. The ‘psychotic feature’ subscale includes items 22, 23, and 24. The ‘insight’ sub-dimension includes items 25, 26, and 27.

#### 2.3.4 Sociodemographic And Personal Data Collection Form

The sociodemographic data collection form consists of 13 items, including the individual’s gender, employment status, marital status, number of children, education level, number of co-habitants, home town, home region, smoking-alcohol-substance use status, and the number of admissions to the psychiatric inpatient services in the last 18 months.

#### 2.3.5 Discharge Readiness Scale 

Cronbach’s alpha for the scale, for which Kaya *et al*. (2018) [26] conducted a Turkish validity and reliability study, was 0.74. The scale consists of 8 items. Answers consist of a 10-point evaluation in the range of 0–10 [26]. The scale allows healthcare providers to determine whether patients are ready for discharge. The scale includes outcomes such as assessment of readiness for discharge from the patient’s perspective, patient safety, satisfaction, and various patient readmissions, healthcare utilization, and mortality.

### 2.4 Ethical Aspects of the Research

During the collection of research data, verbal and written permission was obtained from participating individuals and, when necessary, from their appointed guardians, by informing them about the research, and them signing an ‘Informed Consent Form’. In addition, institutional permission was received from the ethics committee responsible for the adult psychiatry inpatient services of Ankara City Hospital affiliated with the Ministry of Health in Turkey (ethics committee decision no: 23.12.2022-3) and Ankara Yildirim Beyazit University Faculty of Health Sciences ethics committee (ethics committee decision no: 2022-1054, 06.10.2022-14).

### 2.5 Implementation of the Research

The research was conducted in three stages.

#### 2.5.1 First Stage of the Research: Creating the Scale Items and Obtaining Expert Opinions

**Fig. 1.**
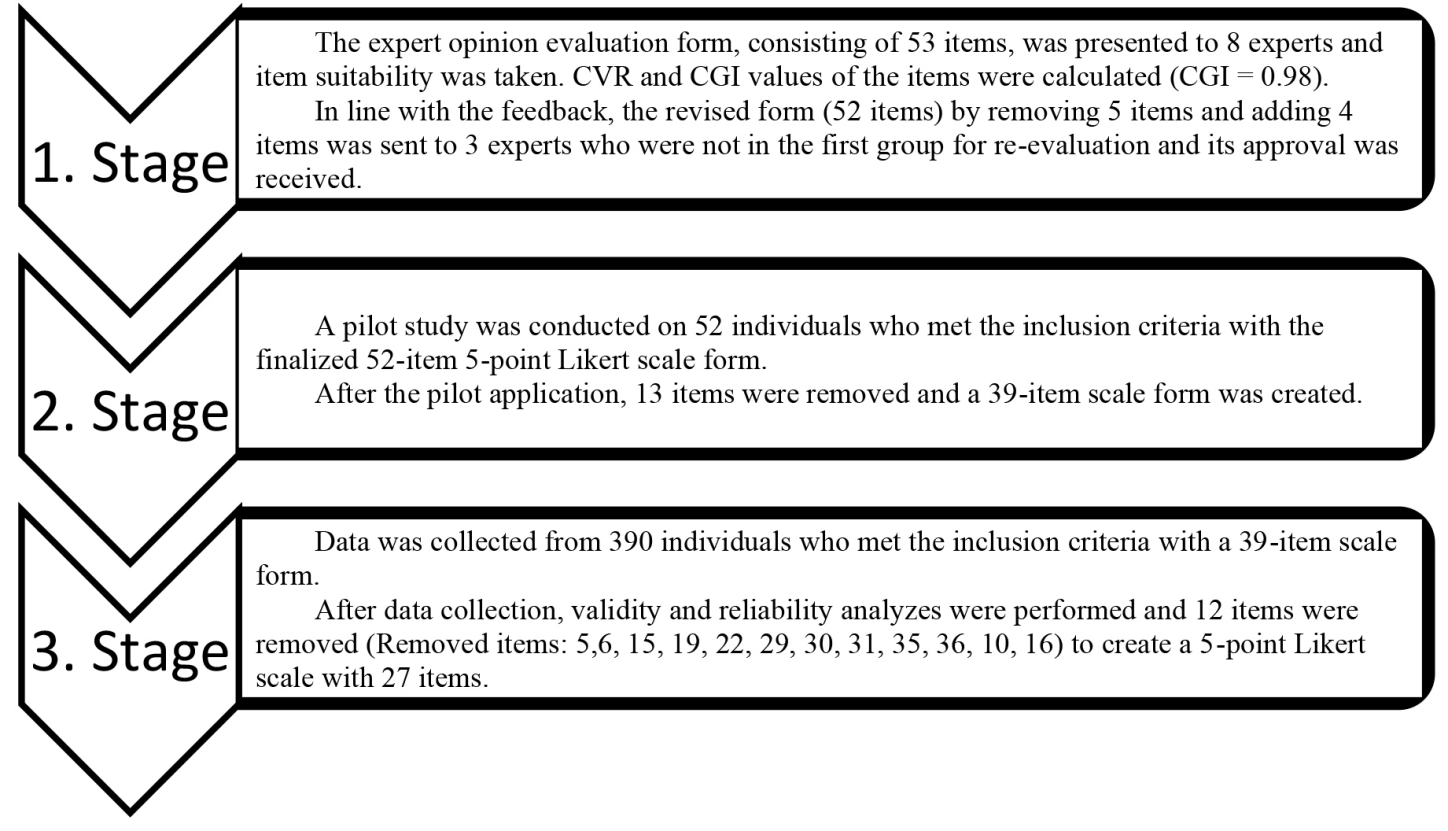
**Research flow chart**. CVR, Scope Validity Ratio; CVI, Scope Validity Index.

First, draft items were determined by the researchers for the development of a scale called RCHRASCPD. These items were developed based on the literature and were then presented to the opinions of at least eight experts in the field of mental health. The items were revised according to expert opinions in line with the literature and three expert opinions were obtained again. The final form of the form was created according to the expert opinions for the second time and was applied to 52 people who agreed to participate in the study for the pilot application and had no obstacles to filling out the form (Fig. 1). After this evaluation, the validity and reliability levels of each item were calculated during the creation of the scale items.

#### 2.5.2 Second Stage of the Research: Conducting the Validity Study

In order to validate the scale, content and construct validity were examined. After content validity was obtained, in the second stage of the research, data collection was carried out using the RCHRASCPD and other relevant scales. At this stage, all the specified data collection forms were applied by the researchers after obtaining permission via the Consent Form from individuals diagnosed with chronic psychiatric diseases who received inpatient treatment at the adult psychiatry inpatient services of Ankara City Hospital between February, 2023 and January, 2024 in Turkey. The flow chart outlining the implementation process of the research is shown in Fig. 1.

2.5.2.1 Content Validity

The Scope Validity Ratio (CVR) and Scope Validity Index (CVI) of the scale evaluated by experts were examined. After completing the scale items with CVR, CVI was calculated using the entire scale. The average of the CVR value of the items in the scale gives the CVI value. CVR (strict) and CVR (relaxed) scores were calculated for each item.

2.5.2.2 Construct Validity

Exploratory Factor Analysis (EFA) and Confirmatory Factor Analysis (CFA) were conducted to determine the construct validity of this scale.

#### 2.5.3 Third Stage of the Research: Reliability Stage

Reliability analysis was considered in three groups. Internal consistency was defined as parallel forms and test-retest reliability. Internal Consistency Reliability: Cronbach’s alpha was used to calculate internal consistency reliability. Parallel Forms Reliability: This type of reliability measures the correlation between the results of two different forms of a test or scale. Both forms were applied at the same time and the results compared. The discharge readiness scale was used as a parallel form. Test-Retest Reliability: The test-retest method was used to evaluate the invariance of the scale over time.

### 2.6 Statistical Analysis

For analysis, Study 2 used SPSS 25.0 and AMOS 28.0 (IBM Corp. (2017). IBM SPSS Statistics for Windows, Version 25.0, Armonk, NY, USA). The exploratory factor analysis removed items with factor loadings below 0.5 or with significant cross-loadings.

Validity analysis: In order to evaluate the validity of the scale, the scope and structure validity were examined. Content validity: Expert opinion was obtained for each item that constitutes the scale. The Scope Validity Ratio (CVR) and Scope Validity Index (CVI) of the scale evaluated by experts were examined. After the scale items were completed with the Content Validity Ratio (CVR), the Content Validity Index (CVI) was calculated using the entire scale. CVR (strict) and CVR (relaxed) scores were calculated for each item. Construct validity: Exploratory Factor Analysis (EFA) and Confirmatory Factor Analysis (CFA) were performed to determine the structural validity of this scale.

Reliability was considered in three groups. These were internal consistency reliability, parallel forms reliability, and test-retest reliability. Cronbach’s alpha was calculated for Internal Consistency Reliability.

### 2.7 Limitations

The fact that data were collected in a single hospital and the number of beds in the inpatient services was insufficient (n = 45) caused the data collection process to take a long time. Some patients who agreed to participate in the study, although they agreed voluntarily, experienced some problems in the process of filling out the survey. These problems resulted in the survey taking longer, some skipping of survey questions, and similar situations. The fact that these situations caused withdrawal from the study is also among the limitations of the study. In this case, it caused the application period of the study to be extended.

## 3. Results

### 3.1 Content Validity

In *Repeat Clinical Hospitalization Risk Assessment Scale in Chronic Psychiatric Diseases*, 10 items were removed from the scale because they reduced the variance explanation rate and item correlations and reliability rate were less than 0.25. After receiving expert opinions, the content validity rate of the items was calculated. Items smaller than the content validity criterion were removed. The content validity index was calculated as 0.98.

After the content validity was obtained, a pilot application was made to 52 individuals with the draft form. After the items were removed, the item correlation was calculated, the scale was finalized, and a 39-item form was created. The 39-item data collection form was applied to 390 individuals in accordance with the inclusion criteria.

### 3.2 Construct Validity

The Kaiser Meyer Olkin (KMO) value for testing sample adequacy and the Bartlett test findings for testing whether the correlation matrix is ​​an identity matrix are shown in Table 1. While performing EFA, the “Varimax” rotation process was applied because the items fit the dimensions more appropriately.

**Table 1.** **KMO value and Bartlett Sphericity test results of the draft Repeat Clinical Hospitalization Risk Assessment Scale in Chronic Psychiatric Diseases**.

Test Applied	Value Achieved	
Kaiser Meyer Olkin (KMO) Sampling Adequacy	0.833	
Bartlett Test of Sphericity	Approx. Chi-Square (χ^2^)	4918.026
	degrees of freedom	406
	*p*-value	0.00001

As shown in Table 2, the total variance explanation rate is above 60% and at an acceptable level.

**Table 2.** **Exploratory Factor Analysis findings regarding the Repeat Clinical Hospitalization Risk Assessment Scale in Chronic Psychiatric Diseases and its Sub-Dimensions**.

Factors/Substances		Factor Loading	Eigenvalue	Variance Explained (%)
Social Support Sources (Factor 1)				
	33. I love my family.	0.880	5.891	13.06
	32. My family always thinks about my well-being and tries to be good.	0.827		
	38. I know that my family is always my support during my treatment process.	0.779		
	34. My family always pays close attention to my problems.	0.774		
	37. I know that I need my family’s support during my illness.	0.718		
Psychosocial Functioning (Factor 2)				
	26. There is someone with whom I can easily share a secret.	0.842	4.499	9.368
	27. I have people I can ask for help when I encounter a problem.	0.789		
	25. I have people around me that I can trust.	0.673		
	28. I can ask others for help when I encounter a problem.	0.625		
Readiness for Discharge (Factor 3)				
	21. I can handle stress when I encounter it.	0.737	2.35	9.283
	17. In my spare time, I do activities such as watching TV, going out, reading books, walking and listening to music.	0.717		
	20. I can work a regular job.	0.645		
	18. I communicate with my family and friends frequently.	0.642		
	16. I can express myself adequately in social environments.	0.623		
Suicide Risk (Factor 4)				
	4. I have attempted suicide in the past.	0.819	1.656	8.754
	1. I have had suicidal thoughts in the past.	0.797		
	2. I still have suicidal thoughts from time to time.	0.708		
	3. I am hopeless about the future.	0.677		
Treatment Compliance (Factor 5)				
	13. I stop taking my medications when I think I am getting better.	0.801	1.563	8.669
	11. I sometimes forget to take my medications when I am not at home.	0.784		
	12. I need help from my relatives to use my medications regularly.	0.782		
	14. Sometimes I don’t use my medications as my doctor recommends.	0.687		
Psychotic Feature (Factor 6)				
	7. I still hear sounds or see images when there is no one around me.	0.771	1.398	7.902
	8. I still think others will hurt me.	0.757		
	9. I still have difficulty concentrating my thoughts on a topic.	0.716		
	10. I still have thoughts of hitting/harming someone else.	0.601		
Insight (Factor 7)				
	24. I think that receiving inpatient treatment in a psychiatric clinic is and will be good for me.	0.832	1.245	7.107
	38. I know that I need to stay in a psychiatric clinic to receive treatment.	0.787		
	39. I do not think that the treatment given in the psychiatric clinic is useful.	0.725		
Overall varience of scale				64.142

### 3.3 Reliability Analysis

The reliability findings of the draft RCHRASCPD are shown in Table 3. There are 29 items in the risk assessment scale for repeated inpatient hospitalization in chronic psychiatric diseases and Cronbach’s alpha was 83.3%. While establishing the CFA model, items with coefficients below 0.5 were removed from the model. In this context, the item ‘I can express myself adequately in social environments’ from the F3 subscale (0.47) and the item ‘I still have thoughts of hitting/harming someone else’ from the F6 subscale (0.48) were removed from the model.

**Table 3.** **Reliability analysis results of the draft Repeat Clinical Hospitalization Risk Assessment Scale in Chronic Psychiatric Diseases**.

	Number of Items	Cronbach’s Alpha (α)
Repeat Clinical Hospitalization Risk Assessment Scale in Chronic Psychiatric Diseases	29	0.833
Factor 1	5	0.891
Factor 2	4	0.845
Factor 3	5	0.753
Factor 4	4	0.802
Factor 5	4	0.789
Factor 6	4	0.724
Factor 7	3	0.748

The index values obtained as a result of the analysis include criteria that show how well the measurement model fits the data set. The Chi-Square (χ^2^) value (2.042) criterion, which is the most important goodness of fit criterion in CFA, is below 3 and therefore represents a perfect fit. Although the Root Mean Square Error of Approximation (RMSEA) value is not in the perfect fit category of the measurement model, it is within the acceptable fit range (0.052). The Normed Fit Index (NFI) value appears to be below the acceptable fit range (0.88). The Comparative Fit Index (CFI) value is within the acceptable fit range (0.93). Goodness of Fit Index (GFI) and Adjusted Goodness of Fit Index (AGFI) values are within acceptable fit ranges (0.90 and 0.87) (Table 4, Ref. [27]).

**Table 4.** **Fit Index Values and Good Fit Criteria for the Measurement Model of the Draft Scale for Assessing Risk of Repeated Clinical Hospitalization in Chronic Psychiatric Disorders**.

Criteria	Perfect Fit	Acceptable Fit	Model Value
RMSEA	0 < RMSEA < 0.05	0.05 ≤ RMSEA ≤ 0.10	0.052
NFI	0.95 ≤ NFI ≤ 1	0.90 ≤ NFI ≤ 0.95	0.88
CFI	0.97 ≤ CFI ≤ 1	0.95 ≤ CFI ≤ 0.97	0.93
GFI	0.95 ≤ GFI ≤ 1	0.90 ≤ GFI ≤ 0.95	0.90
AGFI	0.90 ≤ AGFI ≤ 1	0.85 ≤ AGFI ≤ 0.90	0.87
χ^2^	χ^2^ <3	χ^2^ <5	2.042

Reference: (Schermelleh-Engel *et al*., 2003) [27]. RMSEA, Rootcx Mean Square Error of Approximation; NFI, Normed Fit Index; CFI, Comparative Fit Index; GFI, Goodness of Fit Index; AGFI, Adjusted Goodness of Fit Index; χ^2^, Chi-Square.

The mean of the total Repeated Clinical Hospitalization Risk Assessment Scale for Chronic Psychiatric Diseases (RCHRASCPD) score is 64.06 and the standard deviation is 18.52. When examined in terms of the Discharge Readiness Scale (DSS), the mean for the ‘personal status’ sub-dimension is 14.40 and the standard deviation is 5.45, the mean for the ‘knowledge’ sub-dimension is 12.39 and the standard deviation is 7.07, the mean for the ‘coping’ sub-dimension is 16.12 and the standard deviation is 4.49, and the mean for the ‘expected value’ sub-dimension is 14.74 and the standard deviation is 6.42. In addition, the total DSS score mean was calculated as 57.65 and the standard deviation was 16.95 (Table 5).

**Table 5.** **Possible Lower and Upper Scores of RCHRASCPD ve DSS for Individuals with Chronic Psychiatric Disorders, Mean and Standard Deviations of Scores**.

Scales		$\bar{x}$ ± SD	Min–Max	Lower and Upper Scores
	Factor 1	9.09 ± 5.28	5–25	5–25
	Factor 2	8.30 ± 4.58	4–20	4–20
	Factor 3	10.26 ± 4.89	5–25	5–25
	Factor 4	9.67 ± 5.17	4–20	4–20
	Factor 5	10.61 ± 5.45	4–20	4–20
	Factor 6	9.63 ± 5.22	4–20	4–20
	Factor 7	6.50 ± 3.63	3–15	3–15
RCHRASCPD-Total		64.06 ± 18.52	29–124	29–145
	Personal Status	14.40 ± 5.45	0–20	0–20
	Knowledge	12.39 ± 7.07	0–20	0–20
	Coping	16.12 ± 4.49	0–20	0–20
	Expected Value	14.74 ± 6.42	0–20	0–20
DSS-Total		57.65 ± 16.95	3–80	0–80

RCHRASCPD, Repeated Clinical Hospitalization Risk Assessment Scale for Chronic Psychiatric Diseases; DSS, Discharge Readiness Scale; SD, Standard Deviation.

When the sociodemographic and personal characteristics of the individuals participating in the study were examined, it was determined that 43.8% of the participants were female. When the age distribution was examined, the rate of individuals between the ages of 25 and 40 years was 55.9%, 69.5% were unemployed, 64.9% were single, 60.3% had children, 32.3% had a university degree or higher, 20.5% lived alone, 95.9% lived at home, 87.7% lived in an urban area, 73.8% smoked, 20.5% drank alcohol, and 5.6% used substances. When the number of psychiatric hospitalizations was considered, 53.8% of the participants were hospitalized once, 27.9% twice, 10.5% three times, 5.1% four times, and 2.6% more times (Table 6).

**Table 6.** **Distribution of Demographic Characteristics of Individuals with Chronic Psychiatric Disorders**.

Variable (n = 390)		N	%
Gender			
	Female	171	43.8
	Male	219	56.2
Age (Mean ± SD = 35.87 ± 10.74)			
	18–25	65	16.7
	26–40	218	5.9
	41 and above	107	27.4
Employment Status			
	Employed	119	30.5
	Unemployed	271	69.5
Marital Status			
	Married	137	35.1
	Single	253	64.9
Having Children			
	Yes	253	60.3
	No	155	39.7
Edecation Level			
	Uneducated	9	2.3
	Primary School	112	28.7
	High School	143	36.7
	University and above	126	32.3
Living Situation			
	Alone	80	20.5
	With Spouse Only	51	13.1
	With Child Only	44	11.3
	With Family/Others	215	55.1
Place of Residence			
	Home	374	95.9
	Institution	9	2.3
	Other	7	1.8
Region			
	Rural	48	12.3
	Urban	342	87.7
Smoking			
	Yes	288	73.8
	No	102	26.2
Alcohol Use			
	Yes	80	20.5
	No	310	79.5
Substance Use			
	Yes	22	5.6
	No	368	94.4
Nımber of Psychiatric Hospitalization			
	Once	210	53.8
	Twice	109	27.9
	Three Times	41	10.5
	Four Times	20	5.1
	Five or More	10	2.6

According to the Kolmogorov Smirnov Test, at a statistical confidence level of 95%, the distributions of the scales and their sub-dimensions do not comply with normal distribution (*p* < 0.05) (Table 7).

**Table 7.** **Normality Test Results for RCHRASCPD and DSS Scales and Subdimensions for Individuals with Chronic Psychiatric Disorders**.

Test Name		Kolmogorov Smirnov			
Scales		Skewness	Kurtosis	Test-Statistic	*p*-value
	Factor 1	1.49	1.67	0.22	0.0001
	Factor 2	1.01	0.13	0.18	0.0001
	Factor 3	0.85	0.00	0.14	0.0001
	Factor 4	0.56	–0.91	0.14	0.0001
	Factor 5	0.38	–1.15	0.14	0.0001
	Factor 6	0.56	–0.93	0.16	0.0001
	Factor 7	0.86	–0.28	0.17	0.0001
RCHRASCPD – Total		0.51	–0.39	0.09	0.0001
	Personal Status	–0.88	–0.19	0.16	0.0001
	Knowledge	–0.59	–1.00	0.15	0.0001
	Coping	–1.31	1.31	0.19	0.0001
	Expected Value	–1.08	–0.06	0.21	0.0001
DSS – Total		–0.59	–0.44	0.09	0.0001

RCHRASCPD, Repeated Clinical Hospitalization Risk Assessment Scale for Chronic Psychiatric Diseases; DSS, Discharge Readiness Scale.

According to the Test-Retest analysis results, all factor scores do not show a statistically significant difference between before and after (*p* > 0.05) (Table 8). When the significant correlations between previous (o) and next (s) measurements were examined, it revealed the consistency of many factors over time and their relationships with each other. While there was a strong positive correlation (r = 0.564, *p* = 0.008) between the previous (F1_o) and next (F1_s) measurements of the first factor, the previous measurement of the second factor (F2_o) showed significant relationships both with its own next measurement (F2_s) (r = 0.698, *p* = 0.000) and with the next measurement of the first factor (F1_s) (r = 0.521, *p* = 0.015). A very strong positive correlation (r = 0.729, *p* = 0.000) was observed between the previous and next measurements of the third factor (F3_o and F3_s), while a similar situation was valid for the fourth factor (between F4_o and F4_s r = 0.879, *p* = 0.000). The previous measurement of the fifth factor (F5_o) established significant positive relationships both with its own next measurement (F5_s) (r = 0.688, *p* = 0.001) and with the next measurement of the sixth factor (F6_s) (r = 0.547, *p* = 0.010). The previous measurement of the sixth factor (F6_o) showed positive correlations with subsequent measurements of the fourth (F4_s) (r = 0.544, *p* = 0.011) and fifth (F5_s) (r = 0.478, *p* = 0.028) factors, while it also exhibited a very strong positive correlation with its own next measurement (F6_s) (r = 0.859, *p* = 0.000). Finally, there was also a very strong positive correlation between the previous and subsequent measurements of the seventh factor (F7_o and F7_s) (r = 0.872, *p* = 0.000) (Table 9). These findings suggest that there is generally strong consistency between the previous and subsequent measurements of the factors and that some factors are significantly correlated with each other.

**Table 8.** **Test-Retest Reliability Coefficients fort the Risk Assessment Scale of Repeated Clinical Hospitalization in Chronic Psychiatric Disorsders**.

			$\bar{x}$ ± SD	t-statistic	*p*-value
Before-After					
*RCHRASCPD*	Factor 1	Befor	10.29 ± 5.21	0.000	1.000
		After	10.29 ± 4.12		
	Factor 2	Before	8.05 ± 2.85	0.498	0.621
		After	7.57 ± 3.33		
	Factor 3	Before	10.76 ± 3.30	–0.315	0.755
		After	11.10 ± 3.56		
	Factor 4	Before	9.33 ± 5.27	–0.252	0.803
		After	9.76 ± 5.76		
	Factor 5	Before	9.71 ± 3.80	0.087	0.931
		After	9.62 ± 3.25		
	Factor 6	Before	8.38 ± 4.51	–0.669	0.508
		After	9.29 ± 4.26		
	Factor 7	Before	7.38 ± 3.51	–0.374	0.710
		After	7.81 ± 3.89		

**Table 9.** **Correlation Coefficients Between Subdimensions of the Risk Assessment Scale of Repeated Clinical Hospitalization in Chronic Psychiatric Disorsders**.

		F1_s	F2_s	F3_s	F4_s	F5_s	F6_s	F7_s
F1_o	r	0.564	0.360	–0.239	–0.234	0.007	–0.137	0.316
	*p*	0.008	0.109	0.297	0.307	0.977	0.554	0.163
F2_o	r	0.521	0.698	0.044	0.010	0.239	0.011	0.154
	*p*	0.015	0.000	0.851	0.966	0.296	0.962	0.506
F3_o	r	–0.333	–0.028	0.729	0.328	0.388	0.322	–0.015
	*p*	0.141	0.904	0.000	0.146	0.083	0.155	0.947
F4_o	r	0.030	–0.186	0.291	0.879	0.166	0.388	–0.350
	*p*	0.898	0.421	0.200	0.000	0.473	0.082	0.120
F5_o	r	0.041	0.168	0.231	0.383	0.688	0.547	–0.136
	*p*	0.861	0.467	0.313	0.087	0.001	0.010	0.557
F6_o	r	–0.192	–0.189	0.138	0.544	0.478	0.859	–0.092
	*p*	0.406	0.413	0.552	0.011	0.028	0.000	0.690
F7_o	r	0.044	0.096	–0.059	–0.190	–0.258	–0.325	0.872
	*p*	0.850	0.679	0.800	0.408	0.258	0.150	0.000
	N	21	21	21	21	21	21	21

r, correlation coefficient; *p*, *p*-value.

When examined on a gender basis, women had a lower average than men in Factor 6 and this difference was statistically significant (*p* = 0.003 < 0.05). When evaluated according to employment status, significant differences were found between working individuals and non-working individuals in Factor 2, Factor 3, Factor 4, and General RCHRASCPD scores, and the average number of working individuals was lower than the average number of non-working individuals (*p* < 0.05) (Table 10).

**Table 10.** **Comparison of RCHRASCPD Sobscale and Total Scores by Demographic Charecteristics of ındividuals with Chronic Psychiatric Disoreders**.

Variable	Factor		$\bar{x}$ ± SD	F	*p*-value
Age					
RCHRASCPD	Factor 1	18–25	8.77 ± 4.25	2.882	0.057
		26–40	9.63 ± 5.65		
		41 and above	8.18 ± 4.94		
	Factor 2	18–25	8.00 ± 3.78	2.579	0.077
		26–40	8.75 ± 4.81		
		41 and above	7.57 ± 4.49		
	Factor 3	18–25	10.38 ± 4.76	0.314	0.730
		26–40	10.39 ± 5.1		
		41 and above	9.94 ± 4.57		
	Factor 4	18–25	10.68 ± 5.72	1.520	0.220
		26–40	9.53 ± 4.95		
		41 and above	9.36 ± 5.23		
	Factor 5	18–25	10.49 ± 5.24	0.054	0.948
		26–40	10.69 ± 5.53		
		41 and above	10.51 ± 5.47		
	Factor 6	18–25	9.65 ± 5.45	0.008	0.992
		26–40	9.56 ± 5.19		
		41 and above	9.56 ± 4.63		
	Factor 7	18–25	6.48 ± 3.2	0.100	0.905
		26–40	6.56 ± 3.51		
		41 and above	6.37 ± 4.11		
	General RCHRASCPD	18–25	64.75 ± 18.35	1.422	0.243
		26–40	65.11 ± 19.31		
		41 and above	61.51 ± 6.84		
Education Level					
RCHRASCPD	Factor 1	Primary or below	8.46 ± 5.53	1.390	0.250
		High school	9.54 ± 5.39		
		University and above	9.17 ± 4.87		
	Factor 2	Primary or below	8.19 ± 4.97	0.232	0.793
		High school	8.51 ± 4.32		
		University and above	8.17 ± 4.52		
	Factor 3	Primary or below	10.86 ± 5.04	1.304	0.273
		High school	10.02 ± 4.69		
		University and above	9.97 ± 4.97		
	Factor 4	Primary or below	10.12 ± 5.54	0.832	0.436
		High school	9.3 ± 4.81		
		University and above	9.66 ± 5.19		
	Factor 5	Primary or below	11.87 ± 6.09	8.010	0.0001
		High school	10.81 ± 5.35		
		University and above	9.17 ± 4.55		
	Factor 6	Primary or below	10.5 ± 5.33	3.031	0.049
		High school	9.29 ± 4.87		
		University and above	9.01 ± 4.97		
	Factor 7	Primary or below	5.82 ± 3.55	3.526	0.030
		High school	6.62 ± 3.66		
		University and above	7.02 ± 3.59		
	General RCHRASCPD	Primary or below	65.82 ± 20.21	1.211	0.299
		High school	64.23 ± 17.71		
		University and above	62.17 ± 17.68		
Living Station					
RCHRASCPD	Factor 1	Alone	11.9 ± 7.14	11.061	0.0001
		With Spouse Only	7.37 ± 3.65		
		With Child Only	8.73 ± 4.49		
		With Family	8.52 ± 4.54		
	Factor 2	Alone	10.00 ± 5.51	4.806	0.003
		With Spouse Only	7.65 ± 4.4		
		With Child Only	7.75 ± 3.98		
		With Family	7.94 ± 4.23		
	Factor 3	Alone	10.09 ± 5.3	0.401	0.753
		With Spouse Only	10.92 ± 5.12		
		With Child Only	9.95 ± 3.78		
		With Family	10.24 ± 4.9		
	Factor 4	Alone	9.93 ± 5.37	0.438	0.726
		With Spouse Only	10.2 ± 5.25		
		With Child Only	9.11 ± 4.81		
		With Family	9.57 ± 5.16		
	Factor 5	Alone	9.66 ± 5.02	1.149	0.329
		With Spouse Only	10.78 ± 5.55		
		With Child Only	10.39 ± 4.87		
		With Family	10.96 ± 5.69		
	Factor 6	Alone	9.36 ± 5.08	0.609	0.609
		With Spouse Only	9.95 ± 4.2		
		With Child Only	8.73 ± 5.05		
		With Family	9.75 ± 5.06		
	Factor 7	Alone	6.99 ± 3.79	1.411	0.239
		With Spouse Only	6.1 ± 3.22		
		With Child Only	7.14 ± 3.65		
		With Family	6.28 ± 3.65		
	General RCHRASCPD	Alone	67.93 ± 18.64	1.506	0.212
		With Spouse Only	62.92 ± 18.51		
		With Child Only	62.25 ± 18.51		
		With Family	63.26 ± 18.42		
Numbur of Hospitalization					
RCHRASCPD	Factor 1	1	8.53 ± 5.15	2.526	0.081
		2	9.72 ± 5.49		
		3 or more	9.75 ± 5.2		
	Factor 2	1	7.71 ± 4.3	6.196	0.002
		2	8.41 ± 4.86		
		3 or more	9.89 ± 4.63		
	Factor 3	1	9.78 ± 4.48	6.157	0.002
		2	10.02 ± 5.12		
		3 or more	12.07 ± 5.34		
	Factor 4	1	9.24 ± 4.96	7.340	0.001
		2	9.14 ± 4.63		
		3 or more	11.76 ± 6.02		
	Factor 5	1	10.14 ± 5.23	5.569	0.004
		2	10.26 ± 5.4		
		3 or more	12.54 ± 5.83		
	Factor 6	1	8.99 ± 4.78	17.762	0.0001
		2	8.68 ± 4.62		
		3 or more	12.68 ± 5.48		
	Factor 7	1	6.1 ± 3.41	2.783	0.063
		2	6.91 ± 3.7		
		3 or more	7.04 ± 4.05		
	General RCHRASCPD	1	60.51 ± 6.69	19.784	0.0001
		2	63.32 ± 18.63		
		3 or more	75.72 ± 19.05		

When examined according to smoking use, the average Factor 6 score of individuals who smoked was higher than that of non-smokers and this difference was significant (*p* = 0.009 < 0.05). When examined according to alcohol use, Factor 2, Factor 6, Factor 7 and General RCHRASCPD score averages of individuals who used alcohol were higher than those who did not use alcohol, and this difference was significant (*p* < 0.05). When examined in terms of substance use, Factor 2, Factor 3, Factor 4, Factor 5, Factor 6, and general RCHRASCPD score averages of individuals who used substances were higher than those who did not use substances, and this difference was significant (*p* < 0.05) (Table 10).

In terms of education level, there was a significant difference between education levels for Factor 5, Factor 6, and Factor 7 sub-dimensions (*p* = 0.0001, *p* = 0.049, *p* = 0.030 < 0.05) (Table 10).

When examined in terms of the number of inpatient hospitalizations, Factor 2, Factor 3, Factor 4, Factor 5, Factor 6, and general RCHRASCPD scores showed a significant difference according to the number of inpatient hospitalizations (*p* < 0.05) (Table 10). For general RCHRASCPD, the average number of those with one inpatient hospitalization was 60.50, the average number of those with two inpatient hospitalizations was 63.32, and the average number of those with three or more inpatient hospitalizations was 75.72 (Table 10).

## 4. Discussion

In this study, Explanatory Factor analysis determined the KMO value to be 0.833. As a result of the Bartlett Sphericity test, it was determined that there was a significant relationship between the variables. Accordingly, it was concluded that the data were suitable for applying factor analysis [28]. The RCHRASCPD explained 64.142% of the total variance. In multi-factor models, it is considered sufficient for the total variance to be between 40% and 60%. It is considered a valid and strong analysis if the variance is between 50% and 75% [29]. Since the total variance explanation rate is over 50%, it seems to be at an acceptable level [30].

The risk assessment scale for repeated inpatient hospitalization in chronic psychiatric diseases has 29 items and the reliability level is 83.3%. Additionally, when reliability levels were examined on the basis of factors, they vary between 89.1% and 72.4%. As Cronbach’s alpha of the scale and its sub-dimensions is over 70%, it can be said that the reliability analysis findings are at an acceptable level. When looking at the literature, it is stated that an internal consistency value of Cronbach’s alpha coefficient between 0.60 and 0.80 is considered reliable for the scale, and a value between 0.80 and 1.00 indicates that the scale is highly reliable [31].

The fit index values and good fit values of the measurement model of the RCHRASCPD include criteria that show how well the measurement model fits the data set [32]. The index values obtained as a result of the analysis show that the measurement model has an acceptable fit in general, but there are areas of potential improvement.

The minimum score that can be obtained from the RCHRASCPD developed within the scope of this research is 29 and the maximum score is 124. While the increase in the score obtained from the scale indicates that the risk of repeated inpatient hospitalization increases for individuals with chronic psychiatric diseases, it can be said that the decrease in the score received from the scale by the patients in the study indicates a decrease in the risk of repeated inpatient hospitalization. Considering the number of psychiatric hospitalizations within 18 months, 53.8% of the participants admitted once, 27.9% twice, 10.5% three times, 5.1% four times and 2% (n = 6) of them had more hospitalizations. It is stated in the literature that 17.0% of individuals with chronic psychiatric diseases have been hospitalized three times and 29.8% have been hospitalized four times or more [33]. In another similar study, it was reported that 24.2% (n = 64) of individuals with alcohol and substance addiction were readmitted in the first 6 months. In another study, the overall incidence of readmissions was 16.04%; the readmission rate within 30 days was found to be 6.26% and the readmission rate between 31 and 180 days was 9.44% [34].

Factor 2 includes items that question the individual’s psychosocial functionality. An increase in the score means a decrease in functionality. When the findings were examined, it was seen that the number of repeated hospitalizations increased as the score received increased. When the literature was examined, similar findings were found. It has been reported that low functionality of the patient is an important risk factor for repeated hospitalization [9]. In a study examining individuals who were readmitted within 30 days after discharge, the importance of psychosocial functionality was emphasized [35].

Factor 3 includes items related to readiness for discharge. As the score increased, it was seen that the individual was not ready for discharge and the number of repeated hospitalizations increased. Similar findings were found when the literature was examined. A study reported that individuals with repeated hospitalizations need a more comprehensive discharge plan and support for the transition to society after psychiatric hospitalization [35].

Factor 4 includes the treatment noncompliance subscale. When the scores obtained from the scale were examined, it was seen that the number of repeated hospitalizations increased as the individual’s level of non-compliance with treatment increased. In a study examining the risk factors for repeated hospitalization, it was reported that increasing treatment compliance will reduce the risk of repeated hospitalization [9]. Another study showed that rehabilitation services and medication monitoring carried out in Community Mental Health Centers (CMHC) increase compliance with treatment and thus contribute to a decrease in the frequency of hospitalization [34]. It seems that the findings in the literature are contradictory, but parallel findings related to our research appear to be in the majority.

Factor 5 includes the individual’s past suicide history and current suicide risk. When the findings were examined, it was seen that as the number of repeated hospitalizations increased, the risk of suicide and the presence of a past suicide history increased. It is reported in the literature that the risk of rehospitalization is high in individuals with chronic psychiatric diseases who attempt suicide [36]. In another similar study, it was reported that those with a history of suicide attempt and previous hospitalization were hospitalized twice or more [37]. In another study, the rate of rehospitalization due to suicide risk or suicide attempt ranged between 7.96% and 11.24% [38]. In a similar study, suicide risk was found to be a determinant of early hospital admission [39]. In another study, 94.8% of patients who applied to the emergency department attempted suicide 1–3 times in the last year. It was found that 42.2% of patients who presented with a suicide attempt were referred to intensive care, while only 3.7% were admitted to the psychiatric ward [40].

Factor 6 includes items questioning the presence of psychotic symptoms. When the findings were examined, it was seen that the number of repeated hospitalizations increased as the presence of psychotic symptoms increased. When the literature was examined, contradictory findings were found. In one study, the average number of repeated hospitalizations of individuals diagnosed with schizophrenia showing psychotic symptoms was found to be 7.41 [41]. In another study, it was found that individuals with repeated hospitalizations had higher scores related to psychotic symptoms [9]. Another study reported that the findings of people who were hospitalized three or more times in 12 months were accompanied by psychotic symptoms [42]. In another study examining individuals with repeated hospitalizations, it was reported that 23% of individuals had repeated hospitalizations and that the individuals’ findings were accompanied by psychotic symptoms [43]. It is thought that the results of the studies may vary due to factors such as the diversity of psychotic symptoms within themselves, whether individuals have insight into the process, the different levels of coping with symptoms in individuals, and the stage at which individuals are in the treatment process.

## 5. Conclusions

Many studies have been conducted emphasizing the importance of repeated hospitalizations in psychiatric inpatient services. There is a need for studies that contribute to the planning and implementation of interventions aimed at preventing and reducing these repeated hospitalizations. This research aimed to develop a comprehensive scale to be used in this process. We believe that this scale will support the detection of individuals at risk of repeated psychiatric hospitalization and the prevention of hospitalization, as well as reducing the rate of repeated hospitalizations by providing early intervention in the process. The importance of the scale can also be emphasized in terms of contributing to the reduction of the financial burden of repeated hospitalizations on the health system.

### Recommendations

In line with the findings, it was concluded that the RCHRASCPD is a valid and reliable scale. It is recommended to expand the item pool and increase the number of experts in the process of obtaining expert opinions, to add the data collection method of qualitative interviews to the scale development process, and to create a scale that includes more comprehensive risk factors by expanding the number of items. It is also recommended to conduct studies examining the opinions of psychiatric nurses regarding repeated inpatient hospitalizations in chronic psychiatric diseases and to conduct studies that include interventions of psychiatric nurses to prevent repeated inpatient hospitalizations in chronic psychiatric diseases.

Within the scope of basic nursing interventions, nurses contribute to the diagnosis and treatment processes by making observations. Preventing treatment non-compliance, which is among the risk factors for repeated hospitalization, should be targeted in the care plan. Nurses inform patients and their families about the disease and treatment by providing education and helping them to develop their self-care skills. They also provide medication follow-up, encourage regular medication use, and increase treatment compliance by providing information about side effects. Before discharge, the patient’s needs should be determined so that they can live a healthy life at home [44, 45, 46].

Advanced nursing interventions include psychoeducation, crisis intervention, family therapy, and individual therapy. Nurses aim to increase the individual’s and family’s compliance with treatment through psychoeducation. They strengthen social support resources, manage the discharge preparation process, and maintain compliance after discharge. This reduces the risk of suicide by increasing individual coping skills and provides insight into psychotic symptoms, creating opportunities for early intervention [44, 45, 46].

## Availability of Data and Materials

Data were evaluated with SPSS 21.1 Applied Biostatistics. The tax package and applied statistical methods and findings package of the research are stored individually by the authors. The datasets used and/or analyzed during the current study are available from the corresponding author on reasonable request.
